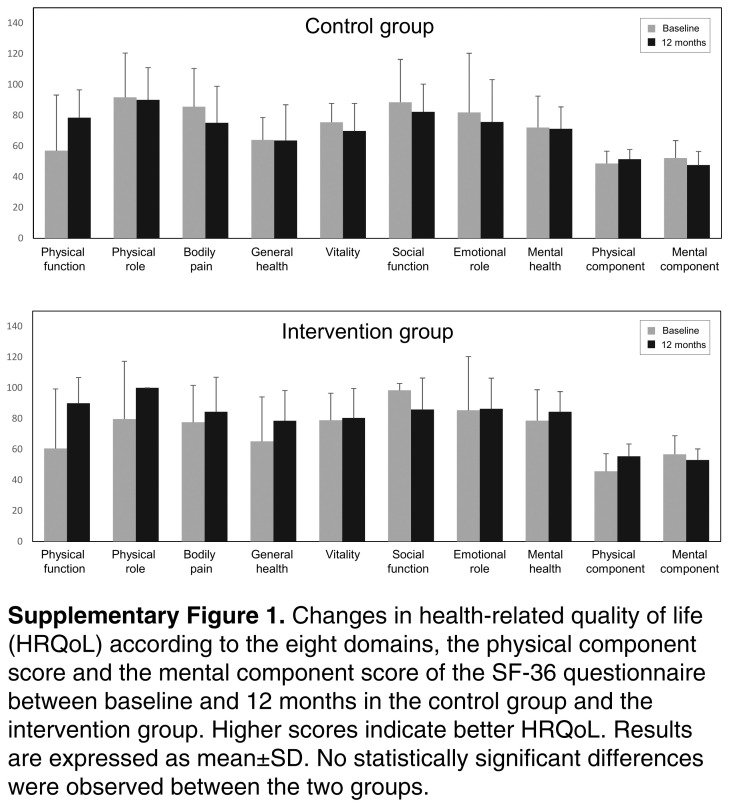# Home exercise, branched-chain amino acids, and probiotics improve frailty in cirrhosis: A randomized clinical trial

**DOI:** 10.1097/HC9.0000000000000443

**Published:** 2024-05-03

**Authors:** Eva Román, Naujot Kaür, Elisabet Sánchez, Maria Poca, Josep Padrós, Maria Josep Nadal, Berta Cuyàs, Edilmar Alvarado, Silvia Vidal, Maria Àngels Ortiz, Elvira Hernández, Rosalía Santesmases, Eulàlia Urgell, Elena Juanes, Andreu Ferrero-Gregori, Àngels Escorsell, Carlos Guarner, Germán Soriano

**Affiliations:** 1 University Nursing School EUI-Sant Pau, Barcelona, Spain; 2 Department of Gastroenterology, Hospital de la Santa Creu i Sant Pau, Barcelona, Spain; 3 Universitat Autònoma de Barcelona, Barcelona, Spain; 4 CIBERehd, Instituto de Salud Carlos III, Madrid, Spain; 5 Institut de Recerca Sant Pau (IR Sant Pau), Barcelona, Spain; 6 Department of Physical Medicine and Rehabilitation, Hospital de la Santa Creu i Sant Pau, Barcelona, Spain; 7 Department of Biochemistry, Hospital de la Santa Creu i Sant Pau, Barcelona, Spain; 8 Department of Pharmacy at Hospital de la Santa Creu i Sant Pau, Barcelona, Spain

## Abstract

**Background:**

Frailty is a predictive factor of hospitalization, falls, and mortality in patients with cirrhosis, regardless of the degree of liver failure. The aim was to analyze whether a multifactorial intervention consisting of home-based exercise, branched-chain amino acids, and a multistrain probiotic can improve frailty in these patients.

**Methods:**

Outpatients with cirrhosis were classified according to the Liver Frailty Index (LFI). Prefrail and frail patients were randomized into 2 groups. The intervention group was assigned to a multifactorial intervention consisting of exercise at home, branched-chain amino acid supplements, and a multistrain probiotic for 12 months. The control group received standard care. All patients were prospectively followed up every 3 months for 1 year to determine LFI, incidence of falls, emergency room visits, hospitalizations, and mortality.

**Results:**

Thirty-two patients were included: 17 patients were assigned to the intervention group and 15 to the control group. In the intervention group, the baseline LFI decreased at 3, 6, 9, and 12 months (*p* = 0.019 for overall change with respect to the control group). The change in LFI (ΔLFI) at 12 months was −0.71 ± 0.24 in the intervention group and −0.09 ± 0.32 in the control group (*p*<0.001). During follow-up, patients in the intervention group had a lower 1-year probability of falls (6% vs. 47%, *p* = 0.03) and emergency room visits (10% vs. 44%, *p* = 0.04) than patients in the control group.

**Conclusions:**

A long-term multifactorial intervention that included exercise at home, branched-chain amino acids, and a multistrain probiotic improved frailty in outpatients with cirrhosis and was associated with a decrease in the incidence of clinical events such as falls and emergency room visits.

## Graphical abstract

**Figure:**
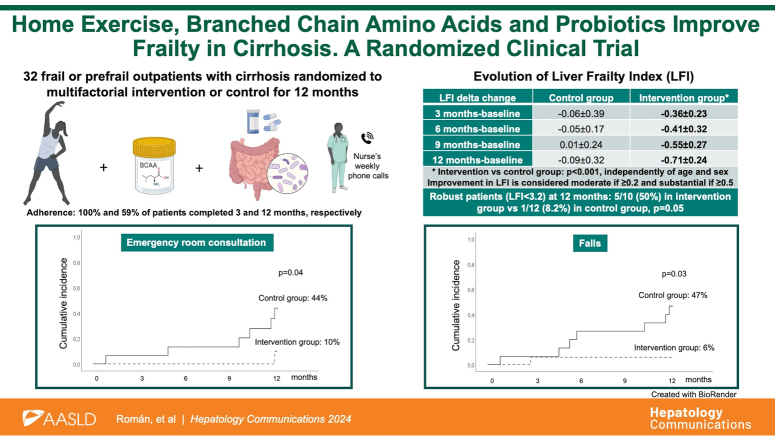

## INTRODUCTION

The life of a patient with cirrhosis of the liver is affected not only by liver failure, portal hypertension, and its complications but also by loss of physiological reserve, manifesting the “frail” clinical phenotype, as described in other chronic diseases.^1–3^ The concept of frailty in cirrhosis goes beyond the degree of liver failure, meaning that it is an expression of dysfunction outside the liver in terms of malnutrition, sarcopenia, and physical and cognitive deterioration.^1–8^

The trajectory from robustness to frailty in patients with cirrhosis is associated with worse clinical outcomes, such as hospitalizations, falls, and mortality, independently of the degree of liver failure.^1–9^ Until recently, however, the only factor considered in the evaluation and decision-making in patients with cirrhosis was the degree of liver failure, and in daily clinical practice, little attention was paid to evaluating and improving frailty.^3^

When considering how to improve frailty in patients with cirrhosis, exercise has proven not only safe but also able to increase functional capacity and quality of life.^10–13^ It has also been demonstrated by means of densitometry and cardiopulmonary exercise testing that a moderate exercise programme in the hospital setting increases muscle mass, decreases fat mass, and improves overall effort time and aerobic exercise time in patients with cirrhosis and overweight.^14^ An alternative modality to site-based exercise programmes is home-based exercise. This modality allows greater accessibility to the exercise programme, including patients with mobility impairment and those living far from the hospital, thus favoring equitable access to exercise training. Other advantages are schedule flexibility, lower costs, and the possibility to include a large number of patients in long-term programmes. Home exercise programmes have recently been evaluated in patients with cirrhosis, with variable results regarding adherence and efficacy.^15–19^

Cognitive impairment in patients with cirrhosis is common and is related to the frailty syndrome and predisposition to fall.^9,20,21^ Falls are frequent both in patients with compensated cirrhosis and decompensated cirrhosis and are a cause of hospital admissions and further deterioration in quality of life.^20–24^ Multiple studies have demonstrated the usefulness of branched-chain amino acids (BCAA) supplements in the prevention of HE, as recently reviewed.^25,26^ Moreover, BCAA can potentiate the beneficial effects of exercise on muscle mass and function.^10,11,26^ Therefore, by ameliorating cognitive function and sarcopenia, BCAA supplementation could help improve frailty in patients with cirrhosis, as recently reported.^27^ Finally, because some probiotics can prevent HE and improve cognitive function and the risk of falls, they may also have an effect on the frailty syndrome in cirrhosis.^28–30^

To our knowledge, it has not been evaluated whether a long-term multifactorial intervention based on exercise, BCAA, and probiotics specifically addressed to prefrail and frail patients with cirrhosis could have a synergistic effect on improving frailty and prefrailty, thereby helping to avoid the consequences of the frailty syndrome such as hospitalizations, falls and death.

This study aimed to determine whether a long-term multifactorial nonpharmacological intervention that included exercise at home, BCAA (branched-chain amino acids), and a multistrain probiotic could improve frailty in patients with cirrhosis.

## METHODS

### Study design

Consecutive patients with cirrhosis from the nursing outpatient office at Hospital de la Santa Creu i Sant Pau, a tertiary care hospital in Barcelona, Spain, were selected by the hepatologists and classified by the nurses according to the Liver Frailty Index (LFI).^1,5^ Prefrail and frail patients were randomized by a nurse to the intervention group or the control group 1:1 using a computer-generated sequence in blocks of 4 and consecutively numbered opaque sealed envelopes. Patients in the intervention group underwent a home exercise program 3 days/week and received BCAA (10 g 30 minutes before each exercise session) and a multistrain probiotic (Vivomixx^®^) 1 sachet every 12 hours. The control group received standard of care without any specific intervention. Robust patients were followed without any specific intervention to assess whether they developed prefrailty or frailty during follow-up. All patients received nutritional counselling.

Patients were prospectively followed every 3 months for 1 year. To better assess the effects of the multifactorial intervention, participants who dropped out of the study were censored after their last visit. At each visit we determined the degree of frailty using the LFI, the risk of falls using the Timed Up & Go test and gait speed, and liver and renal function. Cognitive function was assessed using the Psychometric Hepatic Encephalopathy Score (PHES), and body composition was evaluated by electrical bioimpedance at baseline and at 6- and 12-month visits. Health-related quality of life (HRQoL) was assessed using the Short Form-36 Health Survey (SF-36) questionnaire at baseline and at 12-month visits. Ultrasound quadriceps muscle thickness was determined at baseline and at 6- and 12-month visits in patients from the intervention group.

We recorded adherence, adverse events and their potential relationship with the intervention programme, and the evolution of patients with special emphasis on the incidence of falls, emergency room visits, hospitalizations, and mortality.

### Patient selection

We included male and female patients aged > 18 years old with cirrhosis diagnosed by clinical, analytical, and ultrasonographic criteria or liver biopsy. All participants were conscious and oriented in time and space and able to understand and follow the study indications. Exclusion criteria were patients with poor prognosis (Model for End-stage Liver Disease [MELD] > 25 or expected survival < 6 mo), HCC or other active neoplastic disease, acute or chronic overt HE, neurological disorder hindering performance of the tests, alcohol consumption in the previous 3 months, severe comorbidities, hospitalization in the previous month, contraindications to exercise or probiotic treatment (immunosuppression), and refusal to sign informed consent.

### Outcomes and end points

The main end point was the change in the degree of frailty as evaluated by the LFI during follow-up. Secondary end points were the incidence of clinical events such as falls, hospitalization, emergency room consultation, and mortality. A composite end point including hospitalization, emergency room consultation, or falls was designed. Other secondary end points were muscle function and mass, risk of falls, cognitive function, and HRQoL.

### Evaluation of frailty: LFI

LFI is based on the evaluation of handgrip strength, the ability to get up from and sit on a chair (timed chair stands), and balance. Detailed instructions regarding the performance of the LFI can be found at https://liverfrailtyindex.ucsf.edu/. The LFI has been validated in patients with cirrhosis and is currently the most widely accepted tool to evaluate frailty in these patients. LFI classifies patients with a score of > 4.4 as frail, those with a score of 4.4–3.2 as prefrail, and those with a score of < 3.2 as robust. A clinically significant improvement in LFI is considered moderate if ≥ 0.2 and substantial if ≥ 0.5.^1,5,18^

### Muscle function and body composition were assessed by handgrip strength, electrical bioimpedance, and ultrasound

Muscle function was evaluated by handgrip strength assessed by a dynamometer (KERN MAP 80K1, Akern) following the manufacturer’s instructions. Using the total body impedance analyzer BIA 101 at a signal frequency of 50 kHz and the software BodyGram PRO V.3.0 (Akern, Florence, Italy), we calculated the phase angle and the estimated body compartments after adjusting for age, gender, weight, and height. Measurements were made in a supine position with 4 conventional electrodes: 2 on the wrist and 2 on the ipsilateral foot. Indications before the test were no food or drink in the previous 4 hours, no exercise in the previous 12 hours, an empty bladder, and removal of any jewelry and clothing with metallic elements.^31^ Moreover, in patients in the intervention group, with the patient in the supine position and hip and knee extended, medium thigh circumference was determined using a measuring tape, and thickness of the quadriceps was evaluated by ultrasound (Philips EPIQ Elite: mode B, 8MHz, linear transductor) at the medium thigh and at the limit between the upper third and the medium third of the thigh.

### Risk of falls

The risk of falls was assessed using the Timed Up & Go test and gait speed.^22,32,33^ The Timed Up & Go test measures the time the patient takes to stand up from a chair, walk 3 meters, turn around, walk back, and sit down on the chair without support. Gait speed was measured according to the time taken to walk 5 meters.

### Incidence of falls, hospitalization, emergency room consultation, and mortality

Falls were prospectively assessed at each visit using a specific questionnaire^20,22^ and a review of clinical records. The number of falls, severity of injuries, and health care needed were also recorded. Injuries were categorized as contusion, wound or fracture, and health care needed was classified as primary care, emergency room care, or hospitalization.^20,22^ The incidence of hospitalization, emergency room consultation, and mortality was assessed during visits with patients and relatives and by the review of medical records. We also used a composite clinical end point that included hospitalization, emergency room visits, or fall.

### Cognitive function

Cognitive function was evaluated using the PHES. This neuropsychological battery has been widely used to assess cognitive function in patients with cirrhosis^34^ and was validated for the Spanish population.^35^ We used the computer program of the Spanish Network of Hepatic Encephalopathy (http://www.redeh.org/phesapp/datos.html).

### Health-related quality of life (HRQoL)

The SF-36 questionnaire was administered to all patients in both groups to evaluate HRQoL. SF-36 has been validated for the Spanish population.^36^

### Multifactorial intervention

#### Exercise at home

The exercise program at home was based on previous reports in patients with cirrhosis^16–18^ and our experience with in-hospital exercise programs in these patients.^10,14^ The programme was especially aimed at the rehabilitation of frail patients and was multicomponent. It consisted of aerobic and anaerobic exercises by pedalling (cardiovascular resistance), resistance exercises with dumbbells and elastic bands (muscular strength), flexibility training by stretching, and coordination and balance exercises. Patients performed 3 sessions of 20–30 minutes per week, with progressive increases for up to 45–60 minutes according to tolerance. Each patient in the intervention group was provided with a pedalling or mini-static bicycle (mini-bike DOMYOS 100, Decathlon), weights (dumbbells) and elastic bands (Decathlon), and a watch heart rate monitor (ONRHYTHM 110-Kalenji).

At the beginning of the study, patients were visited by a physician specialized in physical medicine and a physiotherapist. At this visit, they received instruction in the exercise programme. This was individualized according to their physical condition, and they were given the necessary material and written information and instructions regarding the programme. To prevent variceal bleeding, exercises that could significantly increase intraabdominal pressure were avoided. The visit was repeated at 6 months and at the end of the study to check compliance, evaluate possible problems with the programme, and review the exercises.

#### Multistrain probiotic

The composition of the probiotic mixture Vivomixx^®^ (De Simone Formulation) (Mendes SA, Lugano, Switzerland) is detailed in Supplemental material, http://links.lww.com/HC9/A881. Patients from the intervention group took 1 sachet of 4.4 g every 12 hours (450 × 10_^9^_ live bacteria per sachet) throughout the study. We chose this multistrain probiotic because previous studies in patients with cirrhosis have shown its efficacy in preventing HE and improving cognitive function and risk of falls.^28,30^

#### Branched-chain amino acids (BCAA)

In addition to the exercise programme and the probiotic, patients in the intervention group received BCAA supplements (L-leucine, isoleucine, and valine in powder with a 8:1:1 ratio in favor of L-leucine) 10 g 30 minutes before each exercise session throughout the study to enhance the effect of exercise on muscle function and mass^10,11,26,37^ and improve cognitive function.^25^ We chose this dose based on previous data in the literature.^25,27,37^

#### Weekly phone calls

Each week, a nurse from the research team phoned participants in the intervention group to ensure adherence to the multifactorial intervention and to detect possible complications.

### Adherence

Adherence while patients were in the programme was evaluated at each 3-monthly visit through a self-reported written diary showing the exercise sessions performed, by counting the returned empty sachets of probiotics and by examination of returned BCAA jars.

### Adverse events

Adverse events were recorded by patients and relatives and reported at each weekly phone call in the intervention group and at 3-monthly visits in the 2 groups. Moreover, the patients in both groups could call the nurse by phone at the patients’ own initiative. We also reviewed the medical records of patients both in the hospital and in primary health care.

### Statistical analysis

Data are expressed as frequencies, percentages, and mean±SD or median (IQR). Student *t*-test (if normal distribution) or Mann-Whitney test (if non-normal distribution) were used for comparison of quantitative variables between the 2 groups at baseline. The normality of data distribution was assessed by the Shapiro-Wilk test. Fisher test was used to compare qualitative variables and the incidence of clinical events. To compare the overall evolution of the main variable—the LFI—throughout follow-up between the 2 groups, we used linear mixed models, adjusting for baseline values, age, gender, MELD, and body mass index. Missing data were managed by sensitivity analysis with multiple imputations. Linear mixed models were also used to compare the overall evolution of the other continuous variables between the 2 groups. After this primary analysis, a secondary analysis was performed using linear mixed models adjusted for multiple comparisons to evaluate intra-group differences at each specific time point. The results of linear mixed models are expressed as coefficient (95% CI). Pearson or Spearman tests were used for correlations. The actuarial probability that patients presented the clinical events was estimated using Kaplan-Meier curves that were compared by log-rank test. Patients who abandoned the study were censored at the last visit. A 2-sided *p* value < 0.05 was considered statistically significant. Statistical analysis was performed using the programme released in 2020, IBM SPSS Statistics for Windows, version 27.0, IBM Corp, Armonk, NY, United States; and R package (R Core Team, Version 4.3.1, 2023).

### Sample size

There were no data in the literature about the effect of the multifactorial intervention administered in this study on frailty in patients with cirrhosis. Considering the relationship between frailty and functional capacity, we calculated the sample size according to the previously reported improvement in functional capacity assessed by the 6-minute walking test, the 2-minute steps or gait speed in patients with cirrhosis after an exercise and leucine supplementation programme, or after treatment with the multistrain probiotic.^10,30^ Assuming an average improvement of 30% in the intervention group and 5% in the control group, with an alpha error of 5%, a beta error of 20%, and 20% of losses, 30 patients (15 per group) would be needed to demonstrate a significant improvement. We used the computer programme GRANMO, version 7.12, 2012, developed by the Institut Municipal d’Investigació Mèdica (IMIM), Barcelona, Spain.

### Ethical aspects

The study conformed to the Guidelines of the Declaration of Helsinki and Istanbul, followed the good clinical practice recommendations and was approved on 23 August 2019 by the ethical committee at our center (Comitè d’Ètica d’Investigació amb Medicaments), approval number IIBSP-FRA-2019-36, 19/212. All patients gave written informed consent after receiving the appropriate information. The study was registered on ClinicalTrials NCT04243148.

## RESULTS

### Characteristics of patients

**FIGURE 1:**
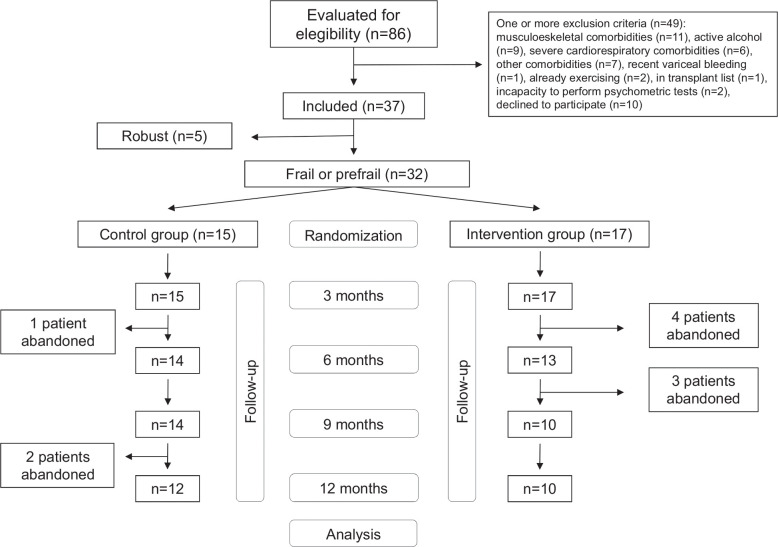
Flowchart.

From March 2021 to July 2022, 86 patients with cirrhosis were evaluated for eligibility at the nursing outpatient office. Forty-nine met one or more exclusion criteria, and 37 patients were finally evaluated with the LFI for inclusion in the study (Figure 1). Five patients were robust according to the LFI and were followed up, undergoing standard of care without any specific intervention. The remaining 32 patients (28 prefrail and 4 frail, 65.7 ± 8.4 y old, women 40.6%, alcohol-associated etiology 68.7%, previously decompensated 81.2%, MELD, median [IQR]: 8 [7–9.7]) were randomized to the control group (n = 15) or the intervention group (n = 17). Table 1 shows that patients’ characteristics at baseline were similar in both groups, with the exception of INR, which was higher in the control group even though MELD scores were similar. There was a correlation between LFI and age (*r* = 0.39, *p* = 0.03), Timed Up & Go test (*r* = 0.67, *p* < 0.001), gait speed (*r* = −0.49, *p* = 0.005), and PHES (*r* = −0.40, *p* = 0.03) at baseline.

**TABLE 1:** Baseline characteristics of patients from the control group and the intervention group

	Control group (n = 15)	Intervention group (n = 17)
Age (y)	68.1 ± 9.3	63.5±7.1
> 65 y (%)	8 (53.3)	8 (47.0)
Male/female (%)	8 (53.3)/7 (46.7)	11 (64.7)/6 (35.3)
Etiology: Alcohol/virus/	9 (60)/1 (6.6)/	13 (76.5)/2 (11.7)/
MASLD/other (%)	2 (13.3)/3 (20)	1 (5.8)/1 (5.8)
Previous decompensations (%)	12 (80)	14 (82.4)
Child-Pugh score, median (IQR)	5 (5–6)	5 (5–5)
MELD score, median (IQR)	8 (7–10)	7.5 (6–9)
Comorbidity index (Charlson), median (IQR)	6 (6–8)	6 (4–7)
Diabetes (%)	4 (26.7)	5 (29.4)
BMI (kg/m^2^)	28.9 ± 3.3	27.5 ± 5.2
PHES, median (IQR)	0 (−1–1)	0 (−1–0)
Previous falls (%)	2 (13.3)	0
LFI	4.07 ± 0.48	4.00 ± 0.33
Components of the LFI:		
handgrip strength (kg)	23.7 ± 7.1	24.8 ± 7.7
timed chair stands (sec)	11.6 ± 3.6	11.3 ± 2.2
balance (sec)	24.7 ± 2.5	25.7 ± 3.1
Timed Up & Go (sec)	12.4 ± 2.3	11.2 ± 1.8
Bilirubin (µmol/L)	18 (14–25)	14 (12–24)
Albumin (g/L), median (IQR)	38 (36–41)	39 (37–40)
INR	1.11 (1.04–1.29)	1.07 (1.00–1.10)^a^
C-reactive protein (mg/L), median (IQR)	3.12 (1.93–5.39)	2.24 (1.09–4.44)
Cystatin C (mg/L), median (IQR)	1.07 (0.93–1.27)	1.05 (0.94–1.34)
Creatinine/cystatin C ratio	0.75 ± 0.15	0.70 ± 0.08
Phase angle (deg)	4.5±1.0	4.3±0.9

*Note:* No statistically significant differences were observed between the 2 groups, except for INR. Results are expressed as percentages, mean±SD or median (IQR).

a *P* = 0.04 with respect to the control group.

Abbreviations: BMI, body mass index; INR, international normalized ratio; LFI, Liver Frailty Index; MASLD, metabolic dysfunction–associated steatotic liver disease; MELD, Model for End-Stage Liver Disease; PHES, Psychometric Hepatic Encephalopathy Score.

### Follow-up and compliance

The mean follow-up was 48.9 ± 9.8 wks in the control group and 40.3 ± 15.2 wks in the intervention group (*p* = 0.23). All patients in both groups reached the 3-month visit. After this point, in the control group, 14/15 (93%) patients completed the 6- and 9-month visits, and 12/15 (80%) completed the 12-month visit. In the intervention group, 13/17 (77%) patients reached the 6-month visit and 10/17 (59%) reached the 9- and 12-month visits. Reasons for abandoning the programme in the intervention group were as follows: 1 patient had a vertebral fracture after a nonfalling accident, 1 moved to another city, another explained that she wanted to dedicate her time to writing, and 4 chose to abandon the programme without giving any specific reason. We found no significant differences between patients in the intervention group that abandoned the programme after the 3-month visit and those who remained in the programme after this visit regarding age, gender, alcohol-associated etiology, Child-Pugh and MELD scores, and baseline LFI or changes in LFI at 3-month visit (Supplemental Table 1, http://links.lww.com/HC9/A881).

While in the programme, intervention group participants self-reported in their written diary that they performed 78.6 ± 28.2% of the exercise sessions throughout the study (> 70%–80% at each 3-monthly time point) (Supplemental Table 2, http://links.lww.com/HC9/A881). Using the cutoff of ≥ 80% of sessions to be considered compliant,^16^ we found 12/17 (70.5%) patients at 3-month visit and 7/10 (70%) at 12 months reported having done ≥ 80% of the exercise sessions. Adherence to BCAA and probiotics was 93.6 ± 17.7% and 95.2 ± 12.6%, respectively.

### Evolution of the liver frailty index (LFI)

Table 2 shows the overall decrease in the LFI during follow-up was more pronounced in the intervention group than in the control group (−0.44 [95% CI −0.76;−0.12], *p* = 0.019, with sensitivity analysis *p* = 0.018). Table 3 shows that the change in LFI (ΔLFI) during follow-up was more marked in the intervention group than in the control group (*p* < 0.001), being similar in women and men and in patients ≥ 65 years old and younger patients. At the study end, only 1/12 (8.3%) patients in the control group was robust, compared to 5/10 (50%) in the intervention group (*p* = 0.05). Of the 5 robust patients included in the study but not randomized, 2 became prefrail during follow-up.

**TABLE 2:** Changes in the LFI in the control group and the intervention group

LFI	Baseline control (n = 15), intervention (n = 17)	3 mo control (n = 15), intervention (n = 17)	6 mo control (n = 14), intervention (n = 13)	9 mo control (n = 14), intervention (n = 10)	12 mo control (n = 12), intervention (n = 10)
Control group	4.07 ± 0.48	4.01 ± 0.77	3.96 ± 0.42	3.96 ± 0.45	3.91 ± 0.52
Intervention group^a^	4.00 ± 0.33	3.62 ± 0.37^b^	3.66 ± 0.36^b^	3.47 ± 0.40^b^	3.31 ± 0.40^b^

a Linear mixed model for overall change comparing intervention group vs control group: −0.44 (−0.76;−0.12), *p*=0.019.

b *P*<0.001 at 3, 6, 9, and 12 mo vs baseline in the intervention group.

Abbreviation: LFI, Liver Frailty Index.

**TABLE 3:** Delta change in the LFI in the control group and the intervention group: (A) in all patients, (B) women, (C) men, (D) aged ≥ 65 years old, and (E) aged < 65 years old

Delta change	Control group	Intervention group^a^
(A) All patients		
3 mo baseline	−0.06 ± 0.39	−0.36 ± 0.23
6 mo baseline	−0.05 ± 0.17	−0.41 ± 0.32
9 mo baseline	0.01 ± 0.24	−0.55 ± 0.27
12 mo baseline	−0.09 ± 0.32	−0.71 ± 0.24^b^
(B) Women		
3 mo baseline	−0.11 ± 0.30	−0.40 ± 0.31
6 mo baseline	−0.14 ± 0.14	−0.48 ± 0.41
9 mo baseline	−0.05 ± 0.24	−0.60 ± 0.34
12 mo baseline	−0.12 ± 0.36	−0.73 ± 0.41
(C) Men		
3 mo baseline	−0.01 ± 0.47	−0.34 ± 0.19
6 mo baseline	0.04 ± 0.16	−0.37 ± 0.29
9 mo baseline	0.07 ± 0.23	−0.52 ± 0.27
12 mo baseline	−0.07 ± 0.23	−0.69 ± 0.17
(D) Age ≥ 65 y old		
3 mo baseline	0.01 ± 0.48	−0.27 ± 0.24
6 mo baseline	−0.13 ± 0.16	−0.47 ± 0.22
9 mo baseline	−0.01 ± 0.22	−0.59 ± 0.27
12 mo baseline	−0.07 ± 0.29	−0.75 ± 0.22
(E) Age < 65 y old		
3 mo baseline	−0.14 ± 0.27	−0.43 ± 0.21
6 mo baseline	0.04 ± 0.14	−0.35 ± 0.39
9 mo baseline	0.05 ± 0.27	−0.51 ± 0.30
12 mo baseline	−0.12±0.37	−0.66 ± 0.27

*Note:* Results are expressed as mean±SD.

a Linear mixed model for overall differences comparing intervention group vs. control group adjusted for gender and age: −0.46 (−0.60; −0.32), *p* < 0.001.

b *P*<0.001 delta 12 mo - baseline vs. delta 3 mo - baseline and *p* = 0.006 vs. delta 6 mo - baseline in the intervention group. Gender and age were not statistically significant in the linear mixed model, suggesting a similar improvement in women and men, and in patients aged ≥ 65 years and those < 65 years old.

Abbreviation: LFI, Liver Frailty Index.

When analyzing the overall changes in the 3 components of the LFI during follow-up, the mean % change for handgrip strength was 3.6 ± 8.7% in the control group and 12.3 ± 20.6% in the intervention group (*p* = 0.12), timed chair stands −9.0 ± 12.5% versus −22.7 ± 11.0% (*p* = 0.001), and balance −4.0 ± 13.4% vs. 1.9 ± 13.8% (*p* = 0.20).

### Risk of falling

Two tests to evaluate functional capacity and the risk of falling, the Timed Up & Go test and gait speed, improved in the intervention group in comparison to the control group (*p* = 0.008 and *p* = 0.02, respectively, for overall change) (Table 4). Patients in the intervention group reached a mean gait speed of 1 meter/second at the study end, meaning a clinically significant improvement in the risk of falls.^33^

**TABLE 4:** Changes in the Timed Up & Go test and gait speed in the control group and the intervention group

	Baseline control (n = 15), intervention (n = 17)	3 mo control (n = 15), intervention (n = 17)	6 mo control (n = 14), intervention (n = 13)	9 mo control (n = 14), intervention (n = 10)	12 mo control (n = 12), intervention (n = 10)
Timed Up & Go test (sec)					
Control group	12.4 ± 2.3	12.0 ± 2.9	12.3 ± 3.0	12.1 ± 2.3	12.0 ± 2.7
Intervention group^a^	11.2 ± 1.8	9.6 ± 1.9^b^	10.2 ± 1.4	10.0 ± 2.1	9.5 ± 1.4^b^
Gait speed (m/sec)					
Control group	0.82 ± 0.16	0.81 ± 0.14	0.81 ± 0.17	0.85 ± 0.16	0.87 ± 0.19
Intervention group^c^	0.88 ± 0.16	0.91 ± 0.12	0.92 ± 0.15	1.01 ± 0.21^d^	1.00 ± 0.10

*Note:* Linear mixed models for overall change comparing intervention group vs. control group:

a Timed Up & Go test −2.09 (−3.53; −0.66), *p* = 0.008.

b Timed Up & Go test: *p* < 0.03 at 3 and 12 mo vs. baseline in the intervention group.

c Gait speed 0.12 (0.03; 0.21), *p* = 0.02.

d Gait speed: *p* = 0.04 at 9 mo vs. baseline in the intervention group.

### Body composition assessed by anthropometry, electrical bioimpedance, and ultrasound

The parameters used to estimate muscle mass—anthropometry, electrical bioimpedance, and ultrasound—did not change significantly in the intervention group (Supplemental Table 3, http://links.lww.com/HC9/A881). In this group, at the end of the study, there was a nonsignificant trend to a decrease in body mass index, an increase in muscle mass, and a decrease in fat mass.

### Cognitive function

Cognitive function evaluated by the PHES did not differ between the 2 groups throughout the study, median (IQR): control group at baseline 0 (−1–1), 6-month 0 (−1–2), and 12-month −0.5 (−2–1); intervention group at baseline 0 (−1–0), 6-month 0 (0–1), and 12-month 1 (−1.5–1), p NS.

### Incidence of falls, hospitalization, emergency room consultation, and mortality

**FIGURE 2:**
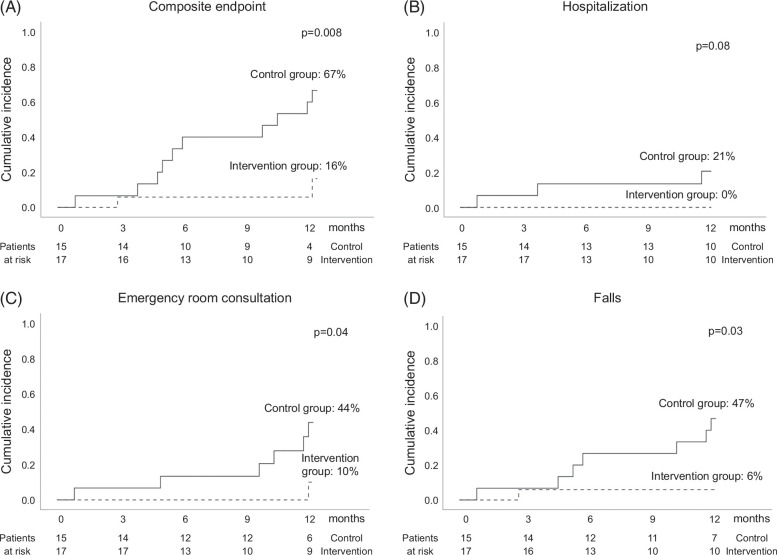
Cumulative incidence of clinical events in the control group and the intervention group: (A) composite end point (hospitalization, emergency room consultation, or falls), (B) hospitalization, (C) emergency room consultation, and (D) falls.

Table 5, Figure 2 and Supplemental Table 5, http://links.lww.com/HC9/A881 show the incidence and probability of the clinical outcomes. Patients in the intervention group presented a lower probability of the composite end point (hospitalization, emergency room visit, or fall) than patients in the control group (16% vs. 67% at 1-year follow-up, *p* = 0.008). They also showed a trend to less hospitalizations (0% vs. 21%, *p* = 0.08) and a statistically significant lower need to go to the emergency room (10% vs. 44%, *p* = 0.04) and to report falls (6% vs. 47%, *p* = 0.03). Regarding falls, 7 patients in the control group presented a total of 8 falls, 3 requiring emergency health care services, 1 for hand fracture, 1 for head trauma, and another for a wound. Only 1 patient in the intervention group presented a fall; this resulted in a contusion, but no specific health care treatment was required. There were no deaths, and no patient needed evaluation for liver transplantation.

**TABLE 5:** Incidence of clinical events during follow-up in the control group and the intervention group

	Control group (n = 15)	Intervention group (n = 17)	*p*
Composite end point (hospitalization, emergency room consultation, or falls), n (%)	10 (66.7)	2 (11.8)	**0.003**
Hospitalization, n (%)	3 (20)	0	0.09
Emergency room consultation, n (%)	6 (40)	1 (5.9)	**0.03**
Falls, n (%)	7 (46.7)	1 (5.9)	**0.01**
Cirrhosis-related events, n (%)	3 (20) 2 variceal bleeding 1 ACLF grade 3 2 spontaneous bacteremia 1 ascites	1 (5.9) HCC	0.34
Mortality, n (%)	0	0	1.00

*Note:* Results are expressed as percentages.

*p* values in bold indicate statistically significant.

Abbreviation: ACLF, acute-on-chronic liver failure.

### Analytical parameters

Table 6 shows there were no significant changes in liver and renal function during follow-up in either group. C-reactive protein decreased in the intervention group in comparison to the control group.

**TABLE 6:** Changes in liver function tests, renal function, creatinine/cystatin C ratio, and CRP in the control group and the intervention group

	Control group			Intervention group		
	Baseline (n = 15)	6 mo (n = 14)	12 mo (n = 12)	Baseline (n = 17)	6 mo (n = 13)	12 mo (n = 10)
Bilirubin (µmol/L)	18 (14–25)	18 (12–33)	19 (15–27)	14 (12–24)	16 (10–21)	15 (10–17)
Albumin (g/L)	38 (36–41)	37 (35–40)	39 (35–41)	39 (37–40)	39 (36–41)	42 (40–44)
INR^a^	1.11 (1.04–1.29)	1.08 (1.02–1.27)	1.12 (1.03–1.12)	1.07^a^ (1.00–1.10)	1.07 (1.00–1.16)	1.06 (1.00–1.10)
Serum sodium (mmol/L)	142.0 ± 2.6	140.8 ± 2.5	140.2 ± 2.1	140.4 ± 1.9	140.5 ± 2.3	139.5 ± 2.0
Creatinine (µmol/L)	71.2 ± 12.8	69.5 ± 12.3	71.3 ± 13.2	72.1 ± 12.3	71.2 ± 9.7	68.6 ± 11.4
Cystatin C (mg/L)	1.07 (0.93–1.27)	1.05 (0.83–1.17)	1.09 (0.87–1.37)	1.05 (0.94–1.34)	1.10 (0.98–1.44)	1.01 (0.90–1.17)
Creatinine/cystatin C ratio	0.75 ± 0.15	0.76 ± 0.15	0.74 ± 0.12	0.70 ± 0.08	0.69 ± 0.06	0.74 ± 0.06
CRP (mg/L)^b^	3.12 (1.93–5.39)	4.80 (2.43–13.17)	3.35 (1.92–7.54)	2.24 (1.09–4.44)	2.55 (1.01–3.59)	1.57 (0.80–2.83)

*Note:* Data are expressed as mean±SD or median (IQR).

a *p* = 0.04 with respect to the control group at baseline.

b −2.88 (−5.48; −0.27), *p* = 0.04 for overall change between the control group and intervention group.

No statistically significant differences were observed in the overall change of the remaining parameters between the 2 groups.

Abbreviations: CRP, C-reactive protein; INR, international normalized ratio.

### Health-related quality of life (HRQoL)

We did not observe any statistically significant differences between the 2 groups in HRQoL measured by the SF-36 questionnaire. However, in the intervention group, there was a trend towards improving the domains of physical function and physical role (Supplemental Figure 1, http://links.lww.com/HC9/A882).

### Adverse events

Supplemental Table 4, http://links.lww.com/HC9/A881, and Supplemental Table 5, http://links.lww.com/HC9/A881, show all the adverse events reported during the study and health care needed. There was a trend of a lower incidence of adverse events per week in the intervention group. No adverse event in the intervention group was considered attributable to any of the components of the programme.

## DISCUSSION

The main finding in this study was that a multifactorial intervention consisting of a home-based exercise programme, BCAA, and a multistrain probiotic improved frailty and decreased the incidence of some adverse outcomes in patients with cirrhosis.

The rationale for using such a multifactorial intervention was the potential synergistic effect between 3 nonpharmacological treatments, each of which has shown on its own to improve frailty or frailty-related parameters in previous studies in patients with cirrhosis. Indeed, exercise programmes in the hospital setting improved muscle mass and function, aerobic fitness, and HRQoL^12–14^; BCAA has been shown to improve muscle mass, frailty,^11,27^ and cognitive function^26^; and some probiotics have been found to ameliorate cognition, physical function and risk of falls.^30^

In comparison with exercise in the hospital setting, there is less evidence regarding the effectiveness of home-based exercise training in cirrhosis, and some reports regarding adherence and efficacy are contradictory.^16–19^ Although home-based training has advantages over site-based exercise, such as less space and staff requirements and lower cost, adherence to home programmes is of concern, with figures ranging from 14% to 75% in 8–12 week programmes.^16–19^ In the present study, all the patients in the intervention group completed at least 3 months of the programme, 77% completed 6 months, and almost 60% reached 12 months. While in the programme, adherence to the home exercise sessions was ≥ 80% at 3-month visits and ≥ 70% at all time points. We therefore consider this compliance satisfactory and likely related to the nurse’s weekly phone calls to potentiate adherence and revise the exercises included in the programme.^38^ Scheduling of phone calls should, therefore, be considered part of the intervention programme. However, beyond phone calls, further measures should be implemented to increase adherence. Such measures could consist of more frequent presential visits, where the particular benefits of the programme could be more discussed in person with each patient, and the use of smartphone applications.^39^

When considering the efficacy of home-based exercise in patients with cirrhosis, while some authors have reported an increase in functional capacity—evaluated either by the 6-minute walking test^19^ or peak aerobic power^16^— others have observed an improvement only in HRQoL.^17,18^ Regarding LFI, few studies have evaluated treatments to improve this index. Lin et al^40^ observed that a home-based exercise programme in transplant candidates improved LFI and was associated with increased survival in those patients achieving an improvement of 0.3. Hernández-Conde et al^11^ reported a decrease in LFI of 0.3 after a 12-week programme of home exercise, an improvement not enhanced by the addition of BCAA. In contrast, other authors have recently observed a decrease of 0.36 in LFI after 16 weeks of BCAA supplementation associated to dietary and exercise counseling, but the decrease was only 0.15 with counseling alone.^27^ Lai et al^18^ did not find a benefit with a 12-week home exercise programme, but adherence was only 14%. In the present study, we found a marked improvement in frailty as assessed by the LFI in the intervention group. The mean improvement in the LFI of 0.36 at 3 months, 0.41 at 6 months, 0.55 at 9 months, and 0.71 at 12 months can be considered clinically moderate (≥ 0.2)^18^ or clinically meaningful (≥ 0.3)^40^ as early as at 3 months, and substantial (≥ 0.5) at 9 and 12 months.^18^ This also suggests an accumulative beneficial effect of the intervention over time. Interestingly, in the control group, only 1 patient (8.3%) was robust at 12 months, but 5/10 participants (50%) in the intervention group reached this condition. In addition, 2/5 (40%) patients classified as robust at the initial evaluation—and therefore not randomized—became prefrail during follow-up, suggesting that interventions to treat and prevent frailty should also be implemented in robust patients with cirrhosis.

When analyzing the percentage of change in the 3 parameters included in the LFI, the improvement in frailty in the intervention group was mainly associated with an increase in handgrip strength and a decrease in timed chair stands, with a less clear improvement in balance. Previous studies have focused on the importance of sarcopenia and the decrease in muscular function as a main feature of frailty syndrome in patients with cirrhosis.^8,9^ It is noteworthy that the increase in muscular function in the intervention group was not associated with an increase in muscle mass as assessed by anthropometry, creatinine/cystatin C ratio, electrical bioimpedance, and ultrasound evaluation of quadriceps thickness. This observation contrasts with previous studies in which exercise at the hospital increased muscle mass in patients with cirrhosis,^10^ and could be due to a lower exercise intensity in the setting of exercising at home. Anyway, the increase in muscular strength suggests an improvement in muscular function, previously described with each one of the 3 treatments combined in the present study.^8,10,26,30^

In the intervention group, in parallel with the improvement in LFI, we observed a significant decrease in C-reactive protein and a nonsignificant trend for serum albumin to increase—both relevant prognostic factors in cirrhosis^41,42^— and also an improvement in functional tests used to assess the risk of falls (ie, the Timed Up & Go test and gait speed). Probably as a consequence of the improvement in frailty and all these related parameters, the probability of clinically relevant events such as emergency room consultations and falls was lower in the intervention group than in the control group. The lower probability of hospitalization and cirrhosis-related events in the intervention group did not reach statistical significance. Our results are in line with those of Lai et al^43^ who reported that the changes in frailty are more relevant than baseline values to predict outcomes. There were no deaths during follow-up in either of the 2 groups, as could be expected considering the relatively preserved liver function of patients included in the study.

In contrast to previous studies using BCAA^25^ or probiotics,^30^ we did not find significant changes in cognitive function assessed by the PHES in either of the 2 groups, probably because no patient presented cognitive impairment at baseline. Also in contrast with previous studies evaluating exercise, BCAA, or probiotics that showed an improvement in HRQoL,^10,12,27,30^ we found only a nonsignificant improvement in the SF-36 domains of physical function and physical role in the intervention group. These findings could be due to the long period elapsed between baseline and the 12-month evaluation of HRQoL; multiple factors other than the multifactorial intervention could have influenced HRQoL. It should be noted that most previous studies assessed HRQoL after shorter interventions, usually of 12 weeks.^10,12,30^

The present study was not designed to elucidate the precise mechanisms involved in the effects of the multifactorial intervention. However, previous studies have reported that exercise improves muscle metabolism and quality,^44^ that BCAA stimulates muscle protein synthesis by activating the mammalian target of the rapamycin (mTOR) pathway,^44^ and that the multistrain probiotic can improve the proinflammatory state and hyperammonemia,^30,45,46^ both factors favoring muscle wasting.^44^

Our study has several limitations. First, the small sample size. However, conducting a long-term study with such a multifactorial intervention is challenging, and even with the low number of patients, we consider the results to be relatively consistent, statistically significant, and clinically relevant. Second, the patients that abandoned the intervention programme could have had a worse baseline condition, resulting in an overestimate of intervention efficacy. Nevertheless, we did not find differences between patients that abandoned the programme after 3 months and those who did not, not even regarding the magnitude of the improvement in the LFI at 3-month visit. Besides, no patients abandoned the programme due to complications of cirrhosis. Third, we cannot be sure about the exact compliance, mainly concerning the exercise-at-home programme, in which adherence relied on patients’ self-reported information. However, the improvement in frailty and muscular strength observed throughout the study suggests that the patients’ high self-reported compliance was reliable. Finally, it was not possible to appraise the individual contribution of each component of the multifactorial intervention and to confirm whether there was a true synergistic effect between the 3 treatments without a comparison with patients on the 3 single treatments. However, previous results suggesting a synergistic effect between exercise and BCAA^10,11,26^ support this hypothesis. In any case, our aim was to test a combined nonpharmacologic intervention consisting of 3 procedures, each of which had previously shown beneficial effects in patients with cirrhosis.

We conclude that a long-term multifactorial intervention based on exercise at home, BCAA, and a multistrain probiotic is feasible, ameliorated frailty in outpatients with cirrhosis, and decreased the probability of emergency room consultations and falls during follow-up. Our results suggest that frailty in cirrhosis is a dynamic and reversible condition that can be improved through nonpharmacological interventions, preferably multifactorial and conducted by a multidisciplinary team. Further studies are needed to confirm whether this improvement can contribute to a better prognosis and quality of life in patients with cirrhosis.

## Supplementary Material

**Figure:**