# Supplementary material for: Home exercise, branched-chain amino acids, and probiotics improve frailty in cirrhosis: A randomized clinical trial
Source: Hepatol Commun. 2024 May 3;8(5):e0443. doi: 10.1097/HC9.0000000000000443 (PMC11073778; doi:10.1097/HC9.0000000000000443)
Supplement: Supplementary file 1 [file hc9-8-e0443-s001.docx]

**SUPPLEMENTARY MATERIAL**

SUPPLEMENTARY METHODS

Multistrain probiotic composition: Vivomixx^®^ (De Simone Formulation) is a probiotic mixture containing the 8 following bacterial strains*: Streptococcus thermophilus* DSM 24731^®^/NCIMB 30438, *Bifidobacterium breve* DSM 24732^®^/NCIMB 30441*, Bifidobacterium longum* DSM 24736^®^/NCIMB 30435 (reclassified *B. lactis*), *Bifidobacterium infantis* DSM 24737^®^/NCIMB 30436 (reclassified *B. lactis*), *Lactobacillus acidophilus* DSM 24735^®^/NCIMB 30442*, Lactobacillus plantarum* DSM 24730^®^/NCIMB 30437*, Lactobacillus paracasei* DSM 24733^®^/NCIMB 30439*, Lactobacillus delbrueckii* subsp. *bulgaricus* DSM 24734^®^/NCIMB 30440 (reclassified *L. helveticus*). Patients from the intervention group took one sachet of 4.4 g every 12 hours (450x10^9^ live bacteria per sachet) throughout the study. The excipient was maltose. The probiotic mixture was manufactured by Danisco-DuPont (Madison, WI, USA) and is currently marketed under the brand Vivomixx^®^ (Mendes SA, Lugano, Switzerland) in Europe and Visbiome^®^ (ExeGi Pharmaceuticals, Rockville, MD) in the USA. Patients were instructed to keep the study product at 4ºC in the refrigerator at home and to take the sachets diluted in one glass of water, milk, or juice at room temperature.

SUPPLEMENTARY TABLES

Supplementary Table 1. Characteristics of the patients in the intervention group who abandoned the programme after the 3-month visit compared to those who remained in the programme after this visit. P NS between the two groups. Results expressed as percentages, mean±SD or median (IQR).

|  | Abandoned  (n=7) | Remained  (n=10) |
| --- | --- | --- |
| Age (yr) | 62.4±5.0 | 64.3±8.5 |
| Male/female (%) | 4 (57.1)/3 (42.9) | 7 (70)/3 (30) |
| Alcohol-related etiology (%) | 6 (85.7) | 9 (90) |
| Child-Pugh score, median (IQR) | 5 (5-6) | 5 (5-5) |
| MELD score, median (IQR) | 8 (7-10) | 7 (6-8.2) |
| Comorbidity index (Charlson), median (IQR) | 6 (4-7) | 6 (4-7) |
| BMI (kg/m^2^) | 27.0±3.4 | 27.5±5.2 |
| Baseline LFI | 3.96±0.18 | 4.01±0.42 |
| Change LFI 3-month vs baseline | -0.40±0.19 | -0.35±0.26 |

MELD: Model for End-stage Liver Disease; BMI: body mass index; LFI: Liver Frailty Index.

Supplementary Table 2. Adherence to the exercise programme, branched chain amino acids (BCAA) and probiotics in the intervention group while patients were in the programme. Expressed as % of exercise sessions performed and doses of BCAA and probiotics taken. Results expressed as mean±SD.

|  | 3 months | 6 months | 9 months | 12 months | Total |
| --- | --- | --- | --- | --- | --- |
| Exercise (%) | 79.1±27.4 | 71.7±30.3 | 80.1±33.5 | 86.9±21.3 | 78.6±28.2 |
| BCAA* (%) | 95.6±18.2 | 89.8±22.2 | 96.7±10.4 | 92.6±16.6 | 93.6±17.7 |
| Probiotics (%) | 93.3±11.1 | 92.5±19.3 | 100±0 | 97.7±7.3 | 95.2±12.6 |

*BCAA: % doses taken with respect to the total sessions of exercise.

Supplementary Table 3. Changes in body composition in the control group and the intervention group: body mass index (BMI), muscle mass, fat mass and phase angle by electrical bioimpedance, medium right thigh circumference and right quadriceps thickness by ultrasound. Results expressed as mean±SD. No statistically significant differences were observed between the two groups in overall change in any parameter.

|  | Control group | | | Intervention group | | |
| --- | --- | --- | --- | --- | --- | --- |
|  | Baseline | 6 months | 12 months | Baseline | 6 months | 12 months |
| BMI (kg/m^2^) | 28.9±3.3 | 28.9±2.8 | 28.7±2.7 | 27.5±5.2 | 26.9±4.6 | 26.6±5.0 |
| Muscle mass (kg) | 29.6±5.6 | 26.9±5.4 | 27.5±6.1 | 25.8±4.7 | 24.4 ±5.2 | 26.9±4.4 |
| Fat mass (kg) | 29.5±6.7 | 30.1±5.5 | 28.6±7.0 | 26.5±8.3 | 26.7±8.1 | 25.1±10.2 |
| Phase angle (º) | 4.5±1.0 | 4.1±0.4 | 4.2±1.1 | 4.3±0.9 | 3.9±1.1 | 4.3±0.6 |
| Medium thigh circumference (cm) | - | - | - | 48.4±7.4 | 48.3±7.2 | 46.8±7.6 |
| Quadriceps thickness (cm)  -Mid  -Upper third | -  - | -  - | -  - | 2.39±0.56  3.07±1.06 | 2.46±0.30  3.04±0.55 | 2.34±0.48  3.05±0.81 |

Supplementary Table 4. Patients with adverse events, severe adverse events and adverse events per week diagnosed during the study in the control group and the intervention group. Results expressed as percentages or mean±SD.

|  | Control group  (n=15) | Intervention group  (n=17) | p |
| --- | --- | --- | --- |
| Patients with adverse events,  n (%) | 11 (73.3) | 9 (53) | 0.29 |
| Patients with severe adverse events, n (%) | 3 (20) | 1 (5.9) | 0.34 |
| Adverse events per week | 0.055±0.073 | 0.017±0.021 | 0.05 |
| Severe adverse events per week | 0.023±0.058 | 0.001±0.004 | 0.29 |

Supplementary Table 5. All the adverse events diagnosed during the study in the control group and the intervention group. **Severe adverse events in bold.** H: required hospitalization; ER: required emergency room consultation.

| Control group  (n=15) | Intervention group  (n=17) |
| --- | --- |
| 2 **variceal bleeding (H)**  1 **ACLF 3 (H)**  2 **spontaneous**  **bacteremia**  **(*E. coli* and *Pseudomonas)* (H)**  1 **ascites (H)**  1 jaundice  1 **intestinal subocclusion (H)**  1 **liver metastases of neuroendocrine**  **tumor**  8 falls (3 ER) (1 hand fracture, 1 head trauma, 1 wound)  2 COVID-19 (1 ER)  1 conjunctivitis (ER)  1 chest pain  1 cold  1 tendinitis (ER)  1 cervical intraepithelial neoplasia (CIN)-1  1 vomiting  1 diarrhea  1 dental abscess  1 dizziness | 1 **hepatocellular carcinoma**  1 enteritis by *Aeromonas*  1 urinary infection  2 leg edema  1 vertebral fracture  1 fall  1 elective surgery hip prosthesis  1 COVID-19  1 cold  1 floaters (ER)  1 lumbar pain |

FIGURE LEGENDS OF SUPPLEMENTARY FIGURES

**Supplementary Figure 1**. Changes in health-related quality of life (HRQoL) according to the eight domains, the physical component score and the mental component score of the SF-36 questionnaire between baseline and 12 months in the control group and the intervention group. Higher scores indicate better HRQoL. Results are expressed as mean±SD. No statistically significant differences were observed between the two groups.
